# Maximal Safe Resection in Glioblastoma Surgery: A Systematic Review of Advanced Intraoperative Image-Guided Techniques

**DOI:** 10.3390/brainsci13020216

**Published:** 2023-01-28

**Authors:** Lapo Bonosi, Salvatore Marrone, Umberto Emanuele Benigno, Felice Buscemi, Sofia Musso, Massimiliano Porzio, Manikon Poullay Silven, Fabio Torregrossa, Giovanni Grasso

**Affiliations:** Neurosurgical Unit, Department of Biomedicine, Neurosciences and Advanced Diagnostics (BiND), University of Palermo, 90100 Palermo, Italy

**Keywords:** glioblastoma, intraoperative imaging technique, 5-ALA, sodium fluorescein, IoMRI, IoUS, neuronavigation, DTI-tractography, quality of life

## Abstract

Glioblastoma multiforme (GBM) represents the most common and aggressive central nervous system tumor associated with a poor prognosis. The aim of this study was to depict the role of intraoperative imaging techniques in GBM surgery and how they can ensure the maximal extent of resection (EOR) while preserving the functional outcome. The authors conducted a systematic review following PRISMA guidelines on the PubMed/Medline and Scopus databases. A total of 1747 articles were identified for screening. Studies focusing on GBM-affected patients, and evaluations of EOR and functional outcomes with the aid of advanced image-guided techniques were included. The resulting studies were assessed for methodological quality using the Risk of Bias in Systematic Review tool. Open Science Framework registration DOI 10.17605/OSF.IO/3FDP9. Eighteen studies were eligible for this systematic review. Among the selected studies, eight analyzed Sodium Fluorescein, three analyzed 5-aminolevulinic acid, two evaluated IoMRI imaging, two evaluated IoUS, and three evaluated multiple intraoperative imaging techniques. A total of 1312 patients were assessed. Gross Total Resection was achieved in the 78.6% of the cases. Follow-up time ranged from 1 to 52 months. All studies assessed the functional outcome based on the Karnofsky Performance Status scale, while one used the Neurologic Assessment in Neuro-Oncology score. In 77.7% of the cases, the functional outcome improved or was stable over the pre-operative assessment. Combining multiple intraoperative imaging techniques could provide better results in GBM surgery than a single technique. However, despite good surgical outcomes, patients often present a neurocognitive decline leading to a marked deterioration of the quality of life. Advanced intraoperative image-guided techniques can allow a better understanding of the anatomo-functional relationships between the tumor and the surrounding brain, thus maximizing the EOR while preserving functional outcomes.

## 1. Introduction

Glioblastoma multiforme (GBM) is the most aggressive and frequent primary malignant tumor of the central nervous system in adults [1]. The mean survival rate is 12–15 months following the gold-standard treatment, including maximal safe resection, radiation therapy, and adjuvant chemotherapy [2]. To date, a few therapeutical approaches have been shown to increase the overall survival (OS) of affected patients, although their efficacy still needs to be supported by large clinical trials [3,4,5].

The OS is mainly associated with the extent of resection (EOR). Lacroix et al. and subsequent studies demonstrated that an EOR > 98% of the tumor mass accounted for a better prognosis [6,7,8]. In this context, surgical planning becomes crucial to extend the OS of affected patients [9,10]. Moreover, due to the growing evidence regarding the biologic behavior of glioma neoplasms, the surgical approach aims to extend the resection beyond the contrast-enhanced tumor borders [11]. Therefore, careful evaluation of the tumor characteristics and its relationships with the adjacent parenchyma and white matter bundles is mandatory and currently possible through advanced imaging techniques. These technological advancements have been shown to assist the neurosurgeon to better understand the brain’s anatomo-functional organization, both maximizing the EOR and minimizing postoperative neurological morbidity [12,13].

The connection between EOR and quality of life (QoL) is the cornerstone of the “onco-functional balance”, based on glioma’s surgical resection tailored on the preservation of cortico-subcortical functions instead of the classical oncological boundaries [14,15]. Such a strategy relies on a detailed pre- and postoperative neuropsychological evaluation, along with a thorough knowledge of the functional anatomy of brain networks.

Recently, it has been shown that connectomic data may play a critical role, providing highly detailed neuroanatomical and neurofunctional maps of the human brain. This strategy could allow evaluating patients’ QoL through the study of a group of functions called “higher-order cognitive functions”, often underestimated during intraoperative monitoring [16,17]. This paradigm shift leads to a network-based approach to glioma surgery, transforming the traditional lesion-oriented approach of neuro-oncologic surgery into functionally tailored resection [18,19]. This shift is certainly made possible by the exceptional technological progress in the field of neuroimaging. The implementation of intraoperative imaging techniques and the development of new strategies aimed at accurate anatomo-functional, metabolic, genomic, and transcriptomic definition is revolutionizing the surgical management of GBM, increasingly emphasizing the importance of patients’ residual QoL.

This systematic review details the role of advanced image-guided techniques in GBM surgery, focusing on their impact on the EOR while preserving the postoperative patients’ QoL. The authors reviewed the feasibility and effectiveness of the main intraoperative imaging techniques such as Intraoperative MRI (IoMRI), Intraoperative Ultrasound (IoUS), sodium fluorescein (SF), 5-aminolevulinic acid (5-ALA), and neuronavigation with/out DTI-fiber tractography, alone or in association. According to the recently released WHO classification, the glioma nomenclature has been modified. Considering the lack of studies analyzing the relationship between intraoperative technique and clinical outcome in GBM surgery according to the most recent classification, it was necessary to maintain the previous WHO classification for our analysis.

## 2. Materials and Methods

### 2.1. Search of the Literature

**Figure 1:**
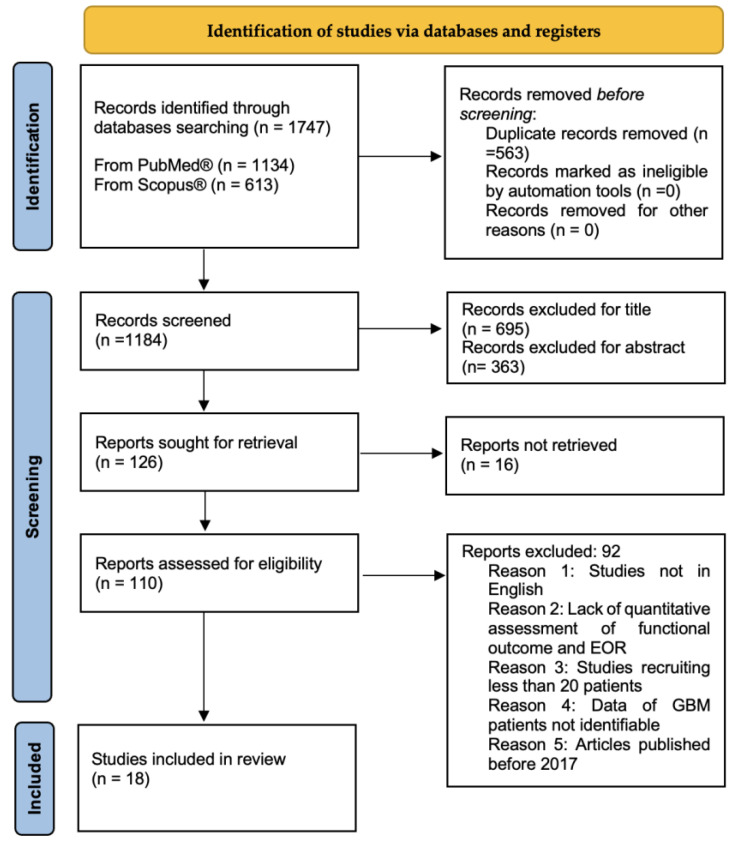
PRISMA flow chart of selection process.

Preferred Reporting Items for Systematic reviews and Meta-analyses guidelines (PRISMA) were followed to conduct this systematic review [20] (Figure 1). We performed a broad systematic literature search in Pubmed/Medline and Scopus for all studies investigating the application of intraoperative imaging techniques in GBM surgery to achieve the best onco-functional balance. We searched for studies published from 2017 up to the 9th of November 2022, using the following MeSH: “Intraoperative MRI (IoMRI)”, “Intraoperative Ultrasound (IoUS), “Sodium Fluorescin”, “5-ALA”, “intraoperative Neuronavigation”, “intraoperative tractography”, “High-grade glioma (HGG)”, “Glioblastoma (GBM)”, “Quality of Life (QoL)”, “Karnofsky Performance Status (KPS)”, and “extent of resection (EOR)”, combined using Boolean operators “AND” and “OR”. Furthermore, we manually screened reference lists of the most relevant systematic reviews and meta-analyses related to this study. Duplicate articles were eliminated using Microsoft Excel 16.37. Since the search combing GBM with the other MeSHs and free text terms revealed limited results, we enlarged the research including in our query “high grade glioma” (HGG). The protocol of this review has been prospectively registered in Open Science Framework and it is available online at https://doi.org/10.17605/OSF.IO/3FDP9 (accessed on 16 December 2022).

### 2.2. Study Selection and Risk of Bias Assessment

The research strategy initially relied on the title and abstract analysis. The article’s full text was retrieved for further investigation if the title and abstract met the inclusion criteria. Two authors (F.B. and P.M.S.) independently assessed eligibility, and differences were resolved with the help of a third author (L.B). We decided to include in the review studies published from 2017, to screen the large amount of data from the literature and, consequently, analyze the most recent evidence. The risk of bias was evaluated using the Risk of Bias in Systematic Reviews (ROBIS) assessment tool [21]. The ROBIS outlines four domains of biases divided into study eligibility criteria assessment, identification and study selection process, data collection and study appraisal evaluation, and synthesis and findings assessment. The data collection process was conducted without using any automated tools. No ethical approval was required for this study.

### 2.3. Eligibility Criteria

The articles were selected according to the following inclusion criteria:

- Full articles in English;
- Clinical studies;
- Studies including patients affected by GBM and/or HGG (studies focusing on HGG were considered only if characteristics of GBM patients were identifiable);
- Age > 18 years old;
- Studies assessing functional outcomes (expressed using standardized scales, i.e., KPS or NANO score);
- Studies assessing EOR.

Exclusion criteria:

- Meta-analysis, reviews, case reports, editorials, technical notes;
- Lack of quantitative assessment of functional outcome and extent of resection;
- Recruited less than 20 patients;
- Article published before 2017.

### 2.4. Data Extraction

Two authors (F.B. and P.M.S.) collected data on study characteristics (authors, publication year, study design, and country), patients’ characteristics (age and sex), type of intraoperative imaging modality used (IoMRI, IoUS, 5-ALA, SF, Neuronavigation and Tractography, Indocyanine green), preoperative mean tumor volume (cm3), number of tumors located into eloquent area (%), surgical and functional outcomes (EOR and KPS), and follow-up duration.

## 3. Results

### 3.1. Study Selection

The search performed yielded 1747 articles. After removing duplicates, the studies screened were 1184. Based on the title and abstract, 1058 articles were excluded and another 16 were not retrieved. A further 92 were not considered due to incompatibility with our eligibility criteria. Full texts of the remaining 18 studies were selected for the analysis. Among the selected studies, 2 were prospective studies, 1 was a case series, 1 Randomized Control Trial (RCT), 1 multicenter cross-sectional study, and 13 retrospective studies. Most studies evaluated multiple intraoperative imaging techniques to achieve maximal EOR while preserving the patient’s neurologic function and QoL. Demographic and study design data are summarized in Table 1.

### 3.2. Study Characteristics

Three studies analyzed the intraoperative use of 5-ALA, alone or in association with other techniques, highlighting its ability to achieve GTR, and thus allowing an increase in OS and progression-free survival (PFS). Picart et al. [37] investigated the impact of 5-ALA florescence-guided surgery associated with IONM (5-ALA group), compared to surgery on white light (control group), in 51 patients affected by GBM located in eloquent areas. Three-month postoperative motor and language deficits rates were similar between groups (5-ALA group: 12.5%, 12.5%; control group: 29.6%, 14.8%) (*p* = 0.180; *p* = 0.990). There were no significant differences in EOR and OS between groups. Interestingly, the 12-month progression-free survival was significantly higher in the 5-ALA group (60%) than in the control group (21%; *p* = 0.006).

Bettag and coauthors [38], in a retrospective series of 20 patients with GBM treated endoscopically, showed that in all the patients, endoscopic fluorescence-guided tumor resection was beyond the contrast-enhanced tumor borders, with a mean postoperative KPS of 89.3. Finally, a multicenter cross-sectional study [30] compared two different groups of GBM patients based on independent variables such as EOR and + surgery (model 1), as well as intraoperative imaging (model 2), and on dependent variables from the European Organization for Research and Treatment of Cancer Quality of Life Questionnaire (EORTC QLQ-C30/BN20). They found a positive correlation between EOR/awake surgery and QoL. On the other hand, in model 2, the impact of intraoperative imaging on QoL has been evaluated, demonstrating that the highest mean scores for functioning and the lowest for symptoms were found in cases where IoMRI was used alone or in combination with 5-ALA.

Eight studies focused on intraoperative use of SF in association with awake surgery [23,31,32,33,34,35,36,39]. Neira’s group [23] showed that with light fluorescence it could be possible to achieve GTR in 84% of patients. In particular, authors divided their cohorts into two groups: “amenable to GTR” and “not amenable to GTR”, based on tumor location. The rate of GTR was higher for patients with a tumor “amenable to GTR” (93.1% of patients). Moreover, they observed a mild reduction in postoperative KPS score (83.3 ± 12.0 preoperative vs. 78.4 ± 13.4 postoperative), especially in the recurrent GBM cohort. Chen et al. [33] showed that DTI with SF improved EOR and postoperative KPS for GBM located in the eloquent area. In their cohort, GTR was achieved in 41 patients (83.7%) while a subtotal resection (STR) was obtained in 8 patients (16.3%). KPS improved in 36 out of 49 patients. Francaviglia and collaborators [35] investigating SF obtained a GTR in 53.2% of the cases and STR in 29.8%. KPS improved in 31.9% of the cases. Ming Lu et al. [36], in their retrospective study, showed a maximal safe extent of resection (>25% FLAIRectomy) by using awake surgery and SF compared to awake surgery alone. Patients who received combined SF and awake craniotomy (AC) resulted in a greater median FLAIR resection percentage (31.90%) as compared with patients treated with AC alone (16.04%). Moreover, although no statistical differences were found in postoperative KPS score between the groups with FLAIR resection above and below 25% of thresholds, the extent of FLAIR GBM abnormality resection was directly related to OS and PFS in a univariate and multivariate analysis. By the way, KPS, PFS, and OS data had to be related to the percentage of tumor located in the eloquent area (58.7%).

Three investigations evaluated the role of IoMRI alone or in association with other intraoperative techniques in extending tumor resection [24,29,30]. Marongiu et al. [24] showed that in the group operated on with the aid of IoMRI, a GTR was achieved in 88.5% of the cases, with an improvement of KPS, at a discharge of 20.5%. In their study, Bassaganyas-Vancell et al. [29] included 118 affected patients according to 5-ALA surgical guidance (5A-group), iMRI (iMRI-group), or both (5A-iMRI-group). An EOR > 90% was more frequent in the 5-ALA group (75%), followed by the IoMRI group (73.7%), and finally the 5A-iMRI-group (71.8%). However, these differences were not statistically significant (*p* value = 0.94), due to the lower rate of tumor located in eloquent areas in the 5-ALA group (26.7%). Further, the occurrence of new neurologic deficits was 21.7%, 20.5%, and 15.8% in 5-ALA, 5-ALA/iMRI, and IoMRI, respectively (*p* value = 0.86).

Two studies focused on the use of IoUS [25,27]. Moiraghi et al. [25], using neuronavigated IoUS (N-IoUS), obtained a gross total resection (GTR) in 61.2% of the patients (vs. 44.8% in Neuronavigated (NN) group). At discharge, the difference between pre- and postoperative KPS was significantly higher for the N-ioUS (*p* < 0.01) compared to the other group.

Different studies [28,33] evaluated the usefulness of intraoperative tractography to maximize EOR while preserving the white matter tracts nearby the tumor. In this regard, Barbagallo et al. [28] reported about recurrent GBM treated with a multimodal approach, including DTI. It has been demonstrated that, with a multimodal approach (neuronavigation with MRI, iCT, 11C-methionine–positron emission tomography (11C-MET-PET), 5-ALA fluorescence, IONM, and N-IoUS), it was possible to reach 100% of GTR with unchanged or improved median KPS over the follow-up period. Chen D. et al. [33] demonstrated that combining preoperative DTI with intraoperative use of SF led to greater EOR for tumor located in the eloquent area: 83.7% of GTR vs. 45.7% in the control group. The authors also assessed the prognosis assessment by evaluating changes in muscle strength and KPS score in the first month after surgery. As a result, they showed that patients treated with preoperative DTI had an improvement in KPS (in 73.5% of cases vs. 47.8% in control group) and less postoperative reduction in muscle strength (20.4% vs. 47.8%) [33]. (Table 2). Three of 18 studies [24,33,38] also enrolled patients with recurrent HGGs in their series, even though the clinical outcome (i.e., KPS) and EOR was not compared between newly diagnosed GBMs and recurrent ones. One of them [33] included only recurrent GBM in its cohort.

### 3.3. Risk of Bias Assessment

The overall methodological quality assessment of this systematic review revealed a moderate-to-high risk of bias (Supplementary Materials). Overall, the quality of the studies retrieved (13 retrospective studies, 2 perspective and 1 RCT) suggests a severe risk of bias in participant selection. Additionally, the high heterogeneity in the patients’ samples, the type of intraoperative imaging techniques, and the lack of reporting of confounders add further concerns. Considering these limitations, the quality of evidence for the included studies was downgraded to low.

### 3.4. Study Synthesis

A total of 1312 patients were evaluated. The mean patient age was 56.5 ± 7.07 years, and a mean M/F ratio was 1.36. On average, GTR was achieved in 78.6% of the evaluated population. An EOR ≥ 90% was obtained in all the analyzed patients. All studies evaluated functional outcomes based on the KPS, while one study used the Neurologic Assessment in Neuro-Oncology (NANO) score. In 77.7% of the studies included (14/18) a variable degree of KPS improvement or its stability, compared with the preoperative period, was recorded. Notably, in 22.2% of the cases (4/18), the functional outcome worsened. The follow-up time was 8.4 ± 15.53 (ranging from 1 to 52 months). Giving the extensive range of follow-up time, it could not be possible to standardize KPS at the same follow-up.

## 4. Discussion

To date, in patients with GBM a maximal surgical resection has been shown to be related with an improvement in OS and PFS. In addition, the EOR beyond the FLAIR’s limits has been demonstrated to be associated with a higher percentage of survivors [40,41]. Despite the best surgical outcome, patients often present a postoperative neurocognitive decline, affecting the QoL.

Thus, it seems clear that there is a need to carefully evaluate which treatment strategy represents the optimal solution, taking into consideration both the OS and subsequent QoL. In this context, this study aims to show a comprehensive review of advanced image-guided intraoperative techniques in GBM surgery.

### 4.1. Fluorescence Techniques

Fluorescence techniques have been used in neurosurgery to obtain a more targeted and safer tumor resection [42]. Four types of intraoperative fluorescence can be distinguished as follows: tissue fluorescence based on passive permeability (i.e., ICG or SF); tissue fluorescence induced by specific metabolic characteristics (5-ALA); auto fluorescence; and, finally, fluorescence derived by fluorescent probes (i.e., near-infrared dyes) [43]. The most promising dyes, frequently used in GBM surgery, are 5-ALA and SF.

#### 4.1.1. Aminolaevulinic Acid

The synthetic amino acid 5-ALA is a compound that, once metabolized, determines the formation of an intermediate fluorescent metabolite known as protoporphyrin IX (PpIX). This molecule accumulates in neoplastic cells in high concentrations [44]. Its value in maximizing EOR in glioma surgery has been highlighted in several studies [45,46,47]. Picart’s group showed that 5-ALA-aided surgery in GBM located in eloquent areas was associated with a significantly higher PFS (*p* value = 0.02), with no modification on the OS or worsening neurological and functional outcomes [37].

Several studies have shown that the use of 5-ALA in GBM surgery leads to an increased rate of GTR along with a good outcome in terms of PFS [48,49,50,51] and OS [52,53,54]. The fluorescence of 5-ALA can usefully differentiate boundaries between lesion core (ALA+), healthy parenchyma (ALA-), and areas of neoplastic infiltration (ALA-PALE), optimizing resection’s limits considering the intratumoral heterogeneity that characterizes GBM [55].

The compound 5-ALA has also been effectively used in recurrent GBM resection [56]. However, the risk of false-positive fluorescence for reactive non-tumor tissue is more remarkable in relapse forms, likely due to altered BBB following adjuvant therapies [57,58]. To be noted, not all types of GBMs uptake 5-ALA [59,60,61]. Suzuki and collaborators [62] found in U251 cell lines an over-expression of the gene encoding Cadherin-13 that would have a negative regulator role in 5-ALA metabolism, making some molecular subtypes of GBM “fluorescence negative” [63].

Interestingly, according to Bonnin et al. [64], different fluorescence intensity could be due to the histological tumor subtype. The Neural GBM subtype has a pattern of reduced fluorescence or even a non-fluorescence signal depending on the genotype analyzed. In this scenario, the association of multiple tracers may offer a viable solution [65]. Della Puppa et al. [53] combined 5-ALA with SF, highlighting the possibility of exploiting two different tracers to maximize EOR, thus improving oncology outcome [66,67].

Finally, Coburger et al. [68] compared the efficacy of IoMRI alone (group 1) versus IoMRI plus 5-ALA fluorescence (group 2) in GBM surgery, showing a significantly higher rate of maximal safe resection in group 2.

#### 4.1.2. Sodium Fluorescein

SF is a fluorescent compound that exploits the altered permeability of the staining brain areas with abnormal cellularity and vascularization. SF is generally thought to bind to blood proteins. Protein-bound SF should be excluded from normal tissue by the BBB because of its dimension, while extravasating in regions where the tumor has compromised this barrier, providing tumor-to-normal contrast [69]. Since its function is based on the altered BBB integrity, SF could provide real-time images of the tumor, especially of its margins. As a matter of fact, a more significant metabolic activity of GBM cells has been seen in such areas [70]. However, it has been reported that BBB’s integrity could be preserved in some GBM, leading to the occurrence of false negatives [71]. Finally, some studies on the biokinetics of SF have shown that this tracer can also accumulate in non-tumor areas, especially where there has been surgical tissue manipulation [42,43].

Microsurgical resection of GBM using SF fluorescence has been associated with an increased GTR rate and OS [72,73,74]. For instance, Chen D et al. [33], in their retrospective cohort study, have evaluated the feasibility and clinical value of magnetic resonance diffusion tensor imaging (MR-DTI) associated with fluorescein in the resection of GBM, demonstrating that the EOR and postoperative KPS were significantly higher in the observation group than in the control group (83.7% vs. 45.7%, respectively; *p* < 0.001 and 73.5% vs. 47.8%, respectively; *p* < 0.029). Similar results were obtained by Raffa et al. [32]. Their study supports the role of the combination of SF-guided resection and Transcranial Magnetic Stimulation (TMS) for the surgical resection of the tumor involving the motor pathway. They compared patients managed by a multimodal approach versus controls, obtaining a higher GTR rate (73.17% vs. 51.22%; *p* = 0.04) and a reduction in cases with new permanent motor deficits (9.75% vs. 29.27%; *p* = 0.04) or worse KPS (12.19% vs. 31.71%; *p* = 0.03).

A safe maximal resection of GBM extended over the contrast-enhanced margins by using SF fluorescence is reported in a study on 32 patients [23]. A GTR was observed in 84% of patients with an average resected volume of 95%. Further studies confirmed the role of SF, alone or in association with different techniques, in gaining the maximum EOR and the best functional outcomes [32,75,76,77,78,79]. Schebesch et al. have recently analyzed 347 patients of a prospective HGG registry, showing significantly more complete resections were achieved in the SF group than in the white-light group (*p* < 0.003) [80].

Finally, it has been shown that an EOR of more than 25% of the FLAIR zone (the so-called FLAIRectomy) for those GBM extending into eloquent areas was safe and associated with a good outcome when the resection was integrated with SF-fluorescence use [36].

### 4.2. Neuronavigation and Tractography

Neuronavigation (NN) is a tool that has gained popularity over the years, giving the opportunity to visualize the surgical scenario in a 3-D model. Years from its first introduction, NN has evolved and integrated with new neuroimaging technologies and new improved algorithms, becoming a useful technique for planning the surgical approach and increasing the accuracy for tumor resection [81]. Some limitations, however, exist and should be taken into consideration. First, intraoperative changes in the surgical field may occur, such as brain shift and brain distortion during surgical maneuvers, limiting the use of NN as a real-time control for the radical resection. Additionally, cyst decompression or deliquoration may provide confounding information in navigational data [82]. This drawback can be overcome through the integration with real-time intraoperative techniques, such as the IoUS or IoMRI. NN has offered outstanding results when associated with brain-mapping techniques such as awake mapping and electrocorticography in the resection of lesions located in the eloquent motor and language areas [83].

Kubben et al. [84] compared the use of NN alone or in association with IoMRI in GBM surgery. The parameters evaluated were the EOR, clinical performance, and the patient’s OS. Interestingly, they found no significant difference between the two imaging techniques. To date, it is also possible to load DTI sequences in the neuronavigator, thus reconstructing tractographic maps that deterministically or probabilistically show three dimensionally the location of eloquent beams.

This tool offers an “in vivo” tracking of the white matter (WM) fiber bundles, providing useful qualitative and quantitative information for the surgical planning on the tracts inside and/or around an intracranial tumor [16,85,86].

The accurate localization of eloquent areas and white matter tracts, such as the corticospinal tract (CST), arcuate fasciculus, and corpus callosum may avoid or reduce the appearance of postoperative neurological deficits [87]. For instance, GBM resection performed in the fronto-parieto-temporal regions of the dominant hemisphere requires the identification of specific functional tracts related to eloquent areas at both cortical and subcortical levels, such as the inferior fronto-occipital fasciculus (IFOF), the inferior longitudinal fasciculus (ILF), the uncinate fasciculus (UF), and the pyramidal tract (PT). Tractography can show the spatial relationship between the tumor and the white matter bundles [88] and estimate the degree of radicality to be reached by evaluating the displacement or the infiltration of the WM fascicles. Their involvement is a strong predictor of the surgical outcome, since the chance of achieving a complete resection is higher when bundles are intact [85].

Interestingly, in a randomized controlled trial of 238 patients (affected by both LGG and HGG comparing NN with or without DTI of the PT), Wu et al. [89] demonstrated that EOR was higher and postoperative neurologic deficits were less frequent with the addition of DTI to the navigational dataset (*p* value < 0.001), especially in HGG group. Furthermore, in patients with motor-eloquent GBM, a novel tractography algorithm called Multi-Level Fiber Tracking (MLFT) has been introduced. This technical refinement adds branches to the pathways previously reconstructed nearby the tumor, improving the reconstruction of the CST compared to conventionally used DTI-based tractography, thus increasing accuracy and safety during surgery [90].

A tractography-related issue is the edema involving the WM around the tumor, which may reduce sensitivity of the technique. In these situations, High-Definition Fiber Tractography (HDFT) may clearly reveal the complex fiber bundles in the perilesional edematous area around the glioma in a three-dimensional way [16]. Further information can be retrieved when tractography is associated with functional Magnetic Resonance Imaging (fMRI) or a 3D ultrasound [87,91,92,93,94].

### 4.3. Intraoperative Ultrasound

IoUS is a safe, non-invasive, intraoperative real-time technique. It is fast, harmless, and cost-effective. B-mode US can provide anatomical information about tumor location [95]; Contrast-Enhanced UltraSound (CEUS) offers practical information about the tumor and its relationships with normal brain parenchyma, having the potential to identify residual tumor volume during surgery. Moreover, CEUS includes valuable information about tumor biological characteristics through direct visualization of its pattern of vascularization [96,97,98,99,100].

It has been suggested that the use of ultrasound in GBM surgery can facilitate complete tumor resection more than standard surgery, maximizing neurological outcome, functional performance, and health-related QoL. Wang et al. [101] have investigated the role of IoUS in improving the survival time of patients with either low- or high-grade gliomas and found that survival rates at 1 and 2 years were significantly increased compared to those of control patients (survival rates at 6 months, 1 year, and 2 years were 83.3% and 93.4%, 43.3% in the observational group, and 59.2%, and 13.3%, and 32.8% in the control group, respectively). As mentioned above, IoUS allows the continuous monitoring of the tumor remnants and the changes after surgical maneuvers, such as brain shift and brain deformation, thus reducing the Residual Tumor Volume (RTV) and increasing the EOR [25,95,102,103,104,105]. In these regards, Mahboob and colleagues [106] found in their meta-analysis that using by IoUS, GTR was obtained in 77% of patients affected by glioma. Incekara et al. [27] observed that complete tumor resection was increased when IoUS was used. Cases in which complete resection was thought to be achieved during the operation corresponded with radiological complete resection in only 11.8% in the standard surgery group and in 46.7% in the IoUS group. Moreover, median operative time with IoUS was not different from standard surgery, thus promoting the use of IoUS as an advantageous intraoperative tool for GBM resection without prolonging operation time. On the other hand, ioUS is an operator-dependent technique and, thus, an effective RTV detection can be achieved by experienced operators. Lastly, IoUS can be associated with other intraoperative techniques, such as NN, 5-ALA, and IoMRI to maximize the extent of contrast-enhancing GBM resection [27,107]. However, further investigations are needed to better understand which association results in the best surgical and functional outcomes.

### 4.4. Intraoperative MRI

IoMRI represents a valuable tool in neurosurgeons’ armamentarium, alone or in association with other techniques [108,109]. While a variety of intraoperative imaging modalities exists, IoMRI provides the highest quality evaluation of surgical resection and assessment of the dynamic changes that occur during surgery [110,111]. Additionally, in providing near real-time information about the dynamic changes occurring during surgery, IoMRI reduces the impact of brain shift phenomenon and improves the accuracy and definition of tumor remnant. In this regard, Hatiboglu et colleagues [112] found that in glioma surgery, even when the resection was considered complete, IoMRI demonstrated an unexpected residual tumor, resulting in additional resection in 47% of cases. Furthermore, IoMRI has been shown to provide useful information to reduce postoperative neurological deficit. In a clinical study, IoMRI improved the EOR by 17.8% and increases the GTR rate by 8.9% up to 73.2%, without additional neurological deficit [113]. Golub et al. [114] have investigated various intraoperative imaging techniques, showing that IMRI and 5-ALA are individually superior to conventional NN for achieving GTR of HGG (*p* < 0.001). Marongiu et al. [24] have analyzed the impact of IoMRI on EOR and KPS, comparing two groups of HGG patients. They demonstrated that the overall GTR for IoMRI group was 88.5%, whereas the non-IoMRI group was 44%. KPS score in IoMRI group was unchanged in 65.4%, improved in 20.5%, and worsened in 14.1% of cases, while in the other group the KPS score was unchanged in 69.5%, improved in 13.8%, and worsened in 16.7%. In another study, Nickel et al. [30] focused on GTR and health-related Quality of Life (HR-QoL), showing a statistically higher GTR when using IoMRI. In addition, they observed a higher score of HR-QoL associated with the use of IoMRI, although the results were not statistically significant. In contrast, it should be considered that ioMRI is a high-cost and time-consuming technique, for reasons that limit the diffusion of this technique.

Though several studies have demonstrated the ability of IoMRI to maximize EOR, to date its impact on the OS has not been fully clarified [115,116,117,118]. Main concerns related to the use of IoMRI are the lack of evidence for a clear benefit and the cost-effectiveness balance.

### 4.5. Future Directions–Digital Biopsy

Due to the continuous evolution in science and technology, new diagnostic and therapeutic tools and strategies have emerged, especially in the neuro-oncological field. Among these promising tools, Raman spectroscopy and confocal laser endomicroscopy have been shown to have a significant impact on glioblastoma surgery.

Raman spectroscopy is a biophotonic tool that can differentiate between different tissue types. It is non-destructive and no sample preparation is required [119,120]. Livermore JL et al. [121] has demonstrated that Raman spectroscopy has excellent sensitivity, specificity, and accuracy in predicting tumor versus normal brain. Moreover, in the glioblastoma cases in which 5-ALA-induced fluorescence was used, the performance of Raman spectroscopy was significantly better than the predictive value of 5-ALA-induced fluorescence (*p* = 0.0009). Certainly, Raman spectroscopy is an extremely innovative option, both for diagnostic and prognostic purposes, as several studies have already shown [122,123,124,125,126], and could become a crucial component in the neurosurgical armamentarium for identifying residual tumors and improving the surgical management of brain tumors [127].

Confocal laser endomicroscopy (CLE) represents a promising technology that provides the real-time histological visualization of living tissue [128,129]. In this context, CLE is an encouraging tool to obtain near real-time intraoperative histological data in neurosurgery. Furthermore, its utility in identifying brain tumor microvasculature, tumor margins, and tumor residual is now well accepted [130]. Höhne J and colleagues [131] evaluated the benefit of CLE in the operative workflow in a series of 12 patients. They found CLE beneficial in terms of high-quality visualization of fine structures and for displaying hidden anatomical details, pointing out its potential to change intracranial tumor surgery. Such evidence also confirms the results obtained by other groups [132,133,134]. However, there are still limitations to be addressed (i.e., the lack of standardized protocols for some of its uses), and further large clinical trials are needed [135].

## 5. Conclusions

In GBM surgery, tumor location strongly influences a radical resection, especially when the tumor involves eloquent areas. Moreover, the ability to infiltrate and spread through WM fiber bundles makes GBM a “whole-brain” pathology. Based on these principles, it is challenging to obtain a complete tumor resection while preserving patient QoL. In the recent years, many intraoperative imaging techniques have blossomed and enriched the surgical armamentarium. Each of them has shown to be of help during the surgical resection, although their limitations must be taken into consideration. The simultaneous use of different techniques identifies the paradigm of the multimodal approach, which, in combining the use of different intraoperative tools, holds promise in reaching a maximal safe resection while preserving neurological functions. Data from this systematic review suggest that multimodal imaging approaches are associated with better surgical and functional outcomes. However, tailored studies focusing on neuropsychological assessment and higher-order cognitive functions are mandatory to better evaluate the link between EOR and QoL. Although the concept of onco-functional balance seems to be well established in low-grade glioma, strong data on GBM and other HGG are still missing. It is well known that the survival of patients affected by HGG is strongly related to surgery, chemo, and radiation therapy and in this scenario the OS is strictly related with EOR. However, an aggressive resection can lead to a dramatic decline in the patient’s cognitive function and QoL, preventing the chance for subsequent adjuvant therapies.

This review has some limitations. First, all studies included in our review do not consider the new WHO classification on gliomas, which incorporates new molecular features. This fact may have led to considering different tumor entities as a single category, predisposing to risks of bias in the assessment of functional outcome and prognosis. Second, the studies included in this systematic review show a great heterogeneity in terms of sample size and imaging techniques used, leading to misinterpretation in the results. Furthermore, most of the included studies are retrospective, implying drawbacks for patient selection and study design. Finally, the evaluation of QoL is related to a simple assessment of the degree of autonomy in the performance of activity of daily life (ADL and BADL), measured by the KPS score. The use of new standardized scales that allow for a comprehensive neuropsychological assessment, including the evaluation of so-called higher cognitive functions (such as the different aspects of memory, comprehension, planning), is essential.

In conclusion, data from this systematic review suggest that in GBM surgery, multimodal imaging approaches are associated with better surgical and functional outcomes. However, the absence of well-tailored studies addressing the onco-functional balance and higher cognitive functions assessment in GBM patients strongly support future well-tailored studies.

## Figures and Tables

**Table 1:** Demographic and study design data included in review.

Author, Year	Country	Study Design	N° Patients	M/F Ratio	Age (Mean)
Sollmann N. et al., 2018 [22]	Germany	Prospective study	60	1.72	47.6
Neira J.A. et al., 2017 [23]	USA	Prospective study	32	1	63.9
Marongiu A. et al., 2017 [24]	Italy	Retrospective study	114	1.15	62.3
Moiraghi A. et al., 2019 [25]	Italy, Switzerland	Case series	60	1.72	55.32
Barbagallo G.M.V. et al., 2021 [26]	Italy	Retrospective study	100	0.88	64
Incekara F. et al., 2021 [27]	Netherlands	Randomized controlled trial	50	1.47	63
Barbagallo G.M.V. et al., 2021 [28]	Italy	Retrospective study	20	1.5	54.5
Bassaganyas-Vancells C. et al., 2019 [29]	Spain	Retrospective study	118	1.51	N/A
Nickel K. et al., 2017 [30]	Germany	Multicenter cross sectional study	170	1.46	55
Luzzi S. et al., 2021 [31]	Italy	Retrospective study	117	1.43	55
Raffa G. et al., 2019 [32]	Italy	Retrospective study	82	1.56	58
Chen D. et al., 2019 [33]	China	Retrospective study	95	0.9	48.5
Hong J. et al., 2019 [34]	China	Retrospective study	82	1.1	51
Francaviglia N. et al., 2017 [35]	Italy	Retrospective study	47	1.13	60.3
Lu M. et al., 2020 [36]	China	Retrospective study	46	1.3	45.1
Picart T. et al., 2017 [37]	France	Retrospective study	51	1.28	47
Bettag C. et al., 2020 [38]	Germany	Retrospective study	20	2	72
Catapano G. et al., 2017 [39]	Italy	Retrospective study	48	1.52	57

**Table 2:** Intraoperative technique, surgical, and functional outcomes included in the study.

Author, Year	Number of Patients with HGG (Number of GBM)	Intraoperative Technique	Preoperative Mean Tumoral Volume (cm^3^)	% of Eloquent Area HGG	Surgical Outcomes (EOR or GTR)	Functional Outcomes	Follow up (mo)
SOLLMANN N. et al., 2018 [22]	46 (21)	AS; nTMS-based DTI-FT; DES; IONM	~26.51	100%	GTR: 80%	Mean KPS: Preop 90; Postop 85; FU 90	3
NEIRA J.A. et al., 2017 [23]	20 (18)	NN; SF	N/A	N/A	GTR: 93.1%	Mean KPS (GBM group only): Preop 85.5; Postop 81.5	N/A
MARONGIU A. et al., 2017 [24]	78 (78) A group 35 (35) B group	ioMRI; NN and DTI (A) vs. NN; preoperative MRI and DTI (B)	28.4 (A) 30.6 (B)	62.8% (A) 30.6% (B)	GTR: 88.5% (A group) 44.4% (B group)	KPS (at discharge): Improved in 20.5% (A group) Improved in 13.8% (B group)	1, 3, 6
MOIRAGHI A. et al., 2019 [25]	60 (51) 31 (N-ioUS groups) 29 (standard NN)	N-ioUS and preoperative mri vs. standard NN and preoperative MRI	36.69 36.21 (N-ioUS) 37.14 (standard NN)	33.3% 31% (N-ioUS) 35.5% (standard NN)	EOR (only GBM group): ~96% NN group ~97% N-ioUS group	ΔKPS (only GBM group): ~6 for the N-ioUS group (*p* < 0.01 for GBM cohort) ~5 for the NN (*p* < 0.01 for GBM cohort)	N/A
BARBAGALLO G.M. V. et al., 2021 [26]	100 (92) A: 48 (45) (=>65 years old) B: 52 (47) (<65 years old)	5-ALA; NN; MEP; SEP; DES; i-CT; NN; IoUS	~42.45	N/A	GTR obtained in: 93.8% (>65 y) 92.3% (<65 y)	KPS: Preoperative: 73.3 (A) vs. 76.5 (B) Postoperative ~74 (A) vs. ~75 (B)	5
INCEKARA F. et al., 2021 [27]	47 (47) 23 in IoUS guided surgery vs. 24 in standard NN	IoUS vs. NN	38.6 (IoUS group) 32.3 (standard NN group)	39.0% (IoUS group) 42.0% (standard NN group)	Median EOR: 97% (IoUS group) 95% (standard NN group)	Mean KPS, (at pre and post operative FU) IoUS group: Preop 90; Postop 90; 90; 90 NN group: Preop 90, Postop 90; 90; 70.	7 weeks, 3, 6
BARBAGALLO G.M.V. et al., 2021 [28]	20 (18)	NN; iCT; 5-ALA; IoUS; IONM; DES; DTI	N/A	100%	EOR: 100% in all except 2 patients	Mean KPS: Preop 80; Postop 75 and then 70 (last FU)	120 (one long survivors)
BASSAGANYAS-VANCELLS C. et al., 2019 [29]	60 (52) 5 ALA group 19 (9) IoMRI group 39 (36) IoMRI + 5-ALA group	5-ALA vs. IoMRI vs. 5-ALA + IoMRI	N/A	26.7% (5-ALA group) 42.1% (IoMRI group) 35.9% (5-ALA + IoMRI group)	EOR => 90%: 75% (5-ALA group) 73.7% (IoMRI group) 71.8% (IoMRI + 5-ALA group)	Mean KPS: 5-ALA: Preop 87.8; Postop 82.6 IoMRI: Preop 91.6; Postop 86.8 5-ALA + IoMRI: Preop 89.7; Postop 85.4	1
NICKEL K. et al., 2017 [30]	170 (123)	AS; 5-ALA; IoMRI (in various combination)	N/A	53.5%	Mean EOR: 95% (5-ALA and IoMRI) 94% (IoMRI) 74% (5-ALA)	Mean KPS: AS group: Preop 45.6 and Postop 64.4 5-ALA + IoMRI: Preop 50.2 and Postop 72.3 Mean postop EORTC: AS: 53.4; IoMRI + 5-ALA: 65.5; IoMRI 63.2; 5-ALA 59.9; no imaging 56.5.	12
LUZZI S. et al., 2021 [31]	117 (95) 54 (32) AR HDFT group 63 (53) standard NN group	AR HDFT + SF vs. NN, brain mapping and SF	12.5 (AR HDFT group) 15.1 (standard NN group)	N/A	EOR: GTR + NTR: 85% (AR HDFT + SF group) GTR + NTR: 65% (control group)	Mean NANO score: AR HDFT group: Preop 5.1 ± 2; Postop 3.8 ± 2 Control group: preop 4.9 ± 2; post op 5.2± 4	12.2
RAFFA G, et al., 2019 [32]	82 (59) 41 (31)–multimodal group 41 (28)–control group	nTMS, SF, IONM (multimodal group) vs. control group	20.3 (multimodal group) 21.1 (control group)	100%	EOR: GTR: 73.17% (group multimodal) GTR: 51.22% (control group)	KPS at 3 months FU: Multimodal group: stable in 58.54%; improved in 29.27% Control group: stable in 53.66%, improved in 14.63%	3
CHEN D. et al., 2019 [33]	95 (44)	DTI and SF (observation group) vs. control	73.8 (in observation group) 69.2 (in control group)	100%	GTR: In 83.6% (observation group) In 45.6% (control group)	KPS Post op: improved in 73.4% (observation group) Post op: improved in 47.8% (control group)	1
HONG J. et al., 2019 [34]	82 (36) 42 (18)–fluorescein group 40 (18)–no fluorescein group	SF vs. no SF group	31.2 (SF group) 30.1 (no SF group)	N/A	EOR: GTR obtained in 85.7% in SF group GTR obtained in 62.5% in no SF group	Mean KPS: SF group: 82 No SF: 75	6
FRANCAVIGLIA N. et al., 2017 [35]	47 (33)	SF	N/A	N/A	EOR: GTR obtained in 53.2%	Mean KPS: Preop: 85.1; Postop 83.4.	10.2
LU M. et al., 2020 [36]	46 (46) 18 (18) (AS group) 28 (28) (AS and SF)	AS + SF vs. AS alone	55.84 (total) 60.47 (AS) 53.13 (AS and SF)	58.7% (total) 30.4% (AS) 28.3% (AS and SF)	EOR: GTR obtained in 100%; a median of 31.90% resection of T2 flair hyperintensity (AS + SF group) GTR obtained in 100%; a median of 16.04% resection of T2 flair hyperintensity (AS alone group)	Mean KPS: AS + SF group: Preop 78.62; Postop 97. AS group: Preop 80; Postop 72.	1
PICART T. et al., 2017 [37]	51 (51) 24 (24) 5-ALA group 27 (27) white light group	5-ALA vs. white light	64.53 (5-ALA group) 78.51 (white light group)	100%	EOR GTR obtained in 67.7% of patients of 5-ALA group GTR obtained in 51.8% of white light group	Mean KPS 5-ALA group: Preop 78.3; Postop 78.5 White light group: Preop 72.6; Postop 74.8	3
BETTAG C. et al., 2020 [38]	20 (20)	5-ALA	29.15	N/A	EOR: GTR obtained in 95% of patients	Mean KPS: Post op 89.3	N/A
CATAPANO G. et al., 2017 [39]	48 (46) 23 (22) SF group 25 (24) control group	SF vs. no SF guidance	30.3 (SF group) 34.9 (control group)	17.3% (SF group) 20% (control group)	EOR: GTR obtained in 82.6% (SF group) GTR obtained in 52% (control group)	Mean KPS: SF group: Preop 80.4; Postop 76.1 Control group: Preop 79.2; Postop 74.8	N/A

Abbreviations and Acronyms: EOR = extent of resection; GTR = Gross total resection (EOR ≥ 95% or higher); STR = subtotal resection (EOR between 25% and 90% of tumor volume); NTR = near total resection (EOR between 90% and 95% of tumor volume); KPS = Karnofsky Performance Scale; ΔKPS = differences between post and preoperative KPS; AS = awake surgery; nTMS = Navigated Transcranial Magnetic Stimulation; DES = direct electrical stimulation; IONM = intraoperative neuromonitoring; DTI = diffusion tensor imaging; DTI FT = DTI fiber tracking; FU = follow-up; NN = neuronavigation; SF = fluorescein sodium; IoMRI: Intraoperative magnetic resonance imaging; N-IoUS, navigated intraoperative ultrasound; MEP = motor evoked potential; SSEP = somatosensory evoked potential; i-CT = computed tomography; AR-HDFT = augmented reality (AR) with diffusion tensor imaging (DTI)-based high-definition fiber tractography; NANO = Neurological Assessment in Neuro-Oncology.

## Supplementary Materials

The following supporting information can be downloaded at https://www.mdpi.com/article/10.3390/brainsci13020216/s1. PRISMA Checklist, ROBIS tool.
